# The effects of biotic treatments on degradation of antimicrobials and coccidiostats in broiler litter used as ruminant feed

**DOI:** 10.1007/s11356-025-36535-9

**Published:** 2025-05-26

**Authors:** Solomon Efriem, Sameer J. Mabjeesh, Chris Sabastian, Malka Britzi

**Affiliations:** 1 The Robert H. Smith Faculty of Agriculture, Food, and Environment, Hebrew University of Jerusalem, P.O. Box 12, Rehovot, 7610001 Israel; 2 National Residue Control Laboratory, Kimron Veterinary Institute, 5025001 Beit Dagan, Israel

**Keywords:** Antimicrobial resistance, Antimicrobials, Coccidiostats, Broiler litter, Short-chain volatile fatty acids (SCVFA), LC/MS/MS

## Abstract

While broiler litter (BL) represents a valuable protein source for ruminant feed, the presence of pharmaceutical residues poses significant concerns for food safety and antimicrobial resistance. Previous research has primarily focused on composting and aerobic digestion of BL, leaving a critical knowledge gap regarding the comparative efficacy of economically viable treatment methods. This study presents the first systematic comparison of three cost-effective treatments—stacking, aerobic, and anaerobic processing—specifically analyzing their impacts on pharmaceutical compound degradation in BL intended for ruminant feed. Using LC/MS/MS analysis, we evaluated the degradation patterns of 29 antimicrobials and coccidiostats under controlled conditions, while simultaneously monitoring key parameters including temperature kinetics, pH fluctuations, short-chain volatile fatty acids (SCVFA), and nutritional content. Our findings revealed that stacking treatment demonstrated superior degradation efficiency for most compounds, achieving > 95% degradation for key antimicrobials and 54.9–74.7% for commonly used coccidiostats. Notably, the stacking method maintained optimal crude protein levels (32–34.8%) while reducing moisture content by 29.5–34.6%. Temperature profiles showed distinct patterns: aerobic treatment reached 60 °C within 24 h, while stacking achieved 50 °C over 2 weeks, both significantly outperforming anaerobic treatment (38 °C). This research provides the first comparative evidence for selecting cost-effective BL treatment methods, offering practical guidelines for agricultural operations to minimize pharmaceutical residues while preserving nutritional value for ruminant feed.

**Supplementary Information:**

The online version contains supplementary material available at 10.1007/s11356-025-36535-9.

## Introduction

According to the American Food and Drug Administration (FDA), 18 classes of antimicrobials are approved for use in food-producing animal (FDA 2011). Antimicrobial dosages vary by compound, the animal being raised, and the country (Bolan et al. 2010). Anti-microbial drugs and coccidiostats commonly used in poultry farming worldwide are critical issues in terms of environmental contamination and on farmers begins from the chicken growing period, as well as when used as ruminant feed and/or fertilizer (Benali and Kudra 2002). In intensive concentrated animal feeding operation systems (CAFOs), litter moisture can reach 40%, pH values between 6.8 and 8, and nutrients found encourage the proliferation of different pathogenic microorganisms. The use of pesticides to protect against flies in the litter environment, residual drugs being excreted, and bad odors caused by the degradation of organic matter by microorganisms are some of the problems associated with poultry litter (Brinsom et al. 1994; Williams et al. 1999; Line 2002).

As reported by the Israel Ministry of Agriculture and Rural Development, Department of Extension Service, in 2021, female and male mix broiler chickens consumed 3737 g of dry feed for up to 35 days. According to the National research council nutrient requirements of poultry (NRC, 1994), broiler chickens are capable of digesting between 85 and 90% of the feed’s dry matter content with some of undigestible feed being excreted as manure. As much as, 800 g of manure and 400 ml of water are excreted by 35–40-day-old birds. The broiler industry extensively utilizes various feed additives, including trace elements such as As, Co, Cu, Fe, I, Mn, Se, and Zn, due to the high-intensity nature of broiler production. A significant proportion of these trace elements and medications consumed by the birds is subsequently eliminated through their excreta, comprising both feces and urine. The variety in litter occurs because of poultry species, animal age, rations, housing type, microorganisms in the gut, and waste management (Perkins et al. 1964; Strasiftak and Juhat 2023).

The use of animal waste as a source of crude protein to animal feed is not a modern invention. Some 3000 years ago, the Chinese grew carp and duck together so as to utilize the duck waste as fish feed (Flachowsky 1997). The co-prophagic phenomena in rabbits, rodents, and pigs were also known in the fourteenth century (Madsen 1939 and Mangold. 1950). The use of poultry litter as ewe feed supplement began in 1939, before the study of Belasco and co-workers on the utilization of uric acid by ruminant microorganisms for the synthesis of protein (Belasco 1954). After the 1954 uric acid and ruminant microorganism study, the first report published by Noland and colleagues opened the door for the use of poultry litter as ruminant feed worldwide (Noland et al. 1955).

The Association of American Feed Control Officials (AAFCO) has approved the use of dried poultry litter as ruminant feed contingent upon pre-treatment and confirmation that it is free of medications and pathogenic microorganisms with AAFCO mandating a 15-day withdrawal period before animal slaughter in cases where medication history is uncertain (AAFCO, 2004); following these recommendations, Israel implemented regulations in 2014 stipulating that ruminants may only be fed treated poultry litter with a mandatory 15-day withdrawal period before slaughter. Additionally, the regulations require analysis of ash content, crude protein percentage, drug residues, pathogenic bacteria count, and copper levels before poultry litter can be utilized as ruminant feed; notably, the regulations include a complete prohibition on feeding poultry litter to dairy cows.

Chemical residues in poultry litter are of concern for ruminant animals when used as feed and in their environment. Yet, for waste manure/broiler litter (BL), regulations that protect against animal toxicity and antibiotic-resistant bacteria stemming from residue drugs is still needed. Research was conducted on the persistence and breakdown of specific antimicrobials and ionophores in BL across various environments, including compost, aerobic digesters, soil, and aquatic systems (Sarmah et al. 2006; Kim et al. 2007; Pugazhendhi et al. 2021). Nevertheless, there is still limited knowledge about how BL processing and the utilization of processed and unprocessed litter as feed for ruminants impacts the breakdown of pharmaceutical substances (Capleton et al. 2006; Hao et al. 2006; Watanabe et al. 2008). A prior ecological investigation revealed that antimicrobial residues in the environment altered soil composition, affected decomposition processes, and impacted the diversity of microorganisms (Keen and Patrick 2013). Furthermore, drug residues are also toxic when found in animal feed and in the environment, such as maduramicin, narasin, and monensin.

The fate of drug residues in BL depends on the bioavailable fraction of the residues and environmental conditions, such as pH, moisture, and organic carbon content, as well as microorganisms (bacteria and fungi) and their interactions with the litter (Swift et al. 1979). Antimicrobials and coccidiostats have low bioavailability and non-absorbed fractions. Therefore, most of it are excreted into the environment. These drugs may persist, may be degraded to non-active compounds, or exist along a gradient of activeness in the litter. One way to increase the value of such treatment is to reduce the moisture content in the litter. To reduce moisture and pathogenic bacteria, litter can be subjected to various treatments. Non-biotic (abiotic) treatment includes hydrolysis, photolysis, oxidation, and reduction processes to degrade drug residues and reduce moisture percentage in litter (Hartlieb et al. 2003). Other biotic processes rely on a decomposition system involving different kinds of microorganisms, some of which do not participate in the biotransformation of drug residues in the litter (Igor and John 2012). The most effective biograding microorganisms were listed in Yang et al. (2020). Biotic treatment involves hydroxylation, decarboxylation, deamination, and epimerization for the biotransformation of drug residues, like tetracyclines and sulphonamides (Tapia et al. 2007; Ma et al. 2021). Other processes, such as volatilization and adsorption, are involved in the degradation of residue drugs in the environment and in BL (Sturini et al. 2012).

Microbial treatments are the most economical and commonly employed BL processing methods. Initially, Israeli regulations mandated BL treatment using a 130 °C drying system. However, this approach proved energy-intensive and costly for both treatment companies and farmers. Consequently, microbial (biotic) treatment systems remain the preferred choice today. In Israel, the most prevalent microbial techniques for processing BL into animal feed include aerobic (forced aeration), anaerobic (ensiling), and stacking treatments. Aerobic (forced aeration) treatment is a microorganisms-based process that occurs in the presence of oxygen. Microorganisms in the litter oxidize organic and nitrogenous compounds. In this process, excess nitrogen is released into the atmosphere as ammonia and reduces litter odor (Tiquia et al. 2002). Stacking treatment of BL is a semi-aerobic treatment that decompose the litter by increasing the porosity hence, supplying nutrition, oxygen, and water for microorganisms (Wang et al. 2015; Zhang and Sun 2016). On the other hand, anaerobic treatment takes place by ensiling the BL directly without adding any organic supplements. Other anaerobic treatments may be conducted by adding different amounts and residues of plants and fruit shells, dairy by-products, and corn silage to increase available organic matter to prompt fermentation (Harmon et al. 1974, 1975; Caswell et al. 1975). The content of water in such treatment should be balanced to 40% of mass to achieve a successful anerobic fermentation. Broiler litter, aging upon treatment, changes the chemical and physical nature of the litter biomass, including reduction of pH (Cressman et al. 2010; Hueso et al. 2012).

Research has been conducted on the impacts of composting and the elimination of antibiotics under controlled temperature conditions, as well as at elevated temperatures (Yudhistra et al. 2019) but not in the case of the abovementioned treatment (i.e., aerobic, anaerobic, and stacking) methods. Since no specific regulatory MRLs exist for poultry litter, monitoring methods must be adapted from existing standards. Using an analytical method with detection limits of 100 ppb, corresponding to typical MRLs established for poultry liver (Efriem et al. 2024), we examined how various treatments affected the breakdown of antimicrobials and coccidiostats. This sensitivity level served two key purposes: monitoring antibiotic residues that could potentially contribute to the development and spread of antibiotic-resistant bacteria from litter to environment, and evaluating the potential toxicity risks of ionophores when treated litter is used as feed for ruminant animals. We analyzed these effects in relation to changes in temperature, pH levels, short-chain volatile fatty acids (SCVFA) release, ash content, and crude protein levels during the application of litter treatments.

## Materials and methods

### Chemicals and reagents and sample collection

A total of 136 kg of BL was used in each round of three different lab-scale treatments to attain a similar treatment process as used in treatment companies. All 136-kg samples were collected from treatment companies’ facilities located at the south of the country, placed in an 80 cm × 100 cm plastic container (Guri Atsmon-Snir Ltd., Barkan, Israel) and transferred to a lab in the Kimron Veterinary Institute, Bet Dagan, Israel.

### Chemicals and reagents

To investigate the degradation of anti-microbial and coccidiostat drug residues, we prepared a mix of 29 compounds used as a spike in BL. The chemical standards for erythromycin, tilmicosin, and oxytetracycline were acquired from Sigma-Aldrich Merck (St. Louis, MO, USA). A2S Analytical Standards Solutions (Saint Jean d'Illac, France) supplied the standards for tylosin, decoquinate, chlortetracycline, and nicarbazin. The following antibiotics were acquired from Toronto Research Chemicals, (Toronto, Canada): teteracycline, ciprofloxacin, danofloxacin, norfloxacin, sulfadiazine, sulfisoxazole, sulfadoxine, sulfadimethoxine, and ampicillin. Glentham (Corsham, Wiltshire, UK) supplied enrofloxacin for the study. Dr. Ehrenstorfer (Augsburg, Germany) provided sulfadimidine, sulfachloropyridazine, doxycycline, amoxicillin, maduramicin, diclazuril, and clopidol. Monensin was acquired from Acros (Geel, Belgium). Narasin was purchased from United States Pharmacopeia (Rockville, MD). HPC Standards (Atlanta, GA, USA) supplied robendine, salinommycin, and sulfachloropyrazine. The purity of all antimicrobial and coccidiostat standards ranged from 97 to 98%, with the exceptions of nicarbazine (90%) and clopidol (95%). J. T. Baker (Deventer, The Netherlands) provided HPLC-grade methanol, acetonitrile, and formic acid, while Sigma-Aldrich Merck supplied EDTA. Fisher Chemicals (Loughborough, UK) was the source for ammonium formate. Analytical grade ethyl acetate, acetonitrile, and methanol were obtained from Bio-Lab (Jerusalem, Israel). A Thermo Scientific device (Long Branch, NJ, USA) was utilized to generate UV/UF-ST deionized water with a resistivity exceeding 18 mΩ/cm. Methanol was used to create stock solutions containing 1000 and 5000 mg kg^−1^ of antimicrobials and coccidiostats, which were then stored at − 20 C.

### Developing a laboratory approach for treating broiler litter using aerobic, anaerobic, and stacking methods

**Fig. 1:**
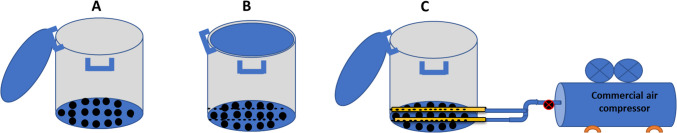
Illustration of the three broiler litter (BL) treatment methods in a lab-scale study. Plastic containers (60-l buckets; Dalas Ltd., Ashdod, Israel) were pre-drilled at the bottom (20-mm-diameter holes) to allow drainage of runoff liquid. Twenty-two kilograms of BL was inserted in all treatments as is without pressing. **A** Stacking treatment, each bucket was open during the 3-week experimental period to allow passive air exchange; **B** anaerobic treatments, buckets were closed to prevent air exchange; however, gaseous was released from the led. The anaerobic treatment lasted for 3 weeks in which short-chain fatty acids were released; **C** aerobic treatment buckets were supplied with two copper tubes (10-mm diameter) from one side at 50-mm distance from the bottom. Copper tubes were equipped with 4-mm holes at the section inside the container. Copper tubes were connected to a commercial air compressor. The treatment lasted 72 h. The compressor supplied air during the first 24 h at 8-min intervals with 4 psi, then the intervals were decreased to maintain the high temperature that was produced from the fermentation

Figure 1 illustrates the three methods used to achieve the BL treatments in lab-scale study. A 60-l container packed with 22 kg BL equipped with an aerobic treatment system was connected to a Balma compressed air system (Torino, Italy) for a period of 72 h. On the initial day, we administered 4 psi of air at three 8-min intervals. For the subsequent 2 days, we decreased the air supply to sustain the self-heating temperature produced by microorganisms. Throughout the treatment, process liquid was discharged through the perforated base of the container. The degradation process was quantified every 24 h (Figures S1 and S2). Concurrently, anaerobic and stacking procedures were conducted in similar vessels each containing 22 kg of BL for a duration of 3 weeks (Figure S3). The initial dry matter contents were adjusted and leveled to 60% for all treatments. The degradation rate was examined every week for the stacking and anaerobic treatments. Drug-free BL was spiked in both experimental groups with initial concentrations (CAo) of antimicrobial drugs sulfisoxazole, amoxicillin, erythromycin, tetracycline, and ciprofloxacin (mix 5) at 5 mg kg^−1^ (ppm low level), 10 mg kg^−1^ (medium level), and 15 and 20 mg kg^−1^ (high levels). Additionally, a combination of 29 antimicrobials and coccidiostats commonly used in poultry farming was added at concentrations of 0.5 mg kg^−1^ (low level), 1 mg kg^−1^ (medium level), and 1.5 and 2 mg kg^−1^ (high level). Each 200 g sample was prepared with four replicates at each concentration level. Broiler litter samples containing spikes were enveloped in 10 × 10 cm^2^ cotton gauze (Top Med, Wuhan, China) and combined with BL mass in containers prior to each experimental treatment. Mix-5- and mix-29-spiked samples were added to the upper and middle BL layers respectively. Mix aliquots were removed before, during, and after treatment for analysis of residues total nitrogen, ash, SCVFA, temperature, and pH. pH was determined by extraction of litter using double-distilled (dd) water (1:10). Dry matter organic matter and ash content were analyzed as described (Hu et al 2009). Crude proteins were analyzed by the total Kjeldahl nitrogen method.

### LC/MS/MS analytical method

The quantification of drug residues was performed using liquid chromatography coupled with tandem mass spectrometry (LC–MS/MS) following our previously published method (Efriem et al. 2024). Analysis was conducted using either an API 4000 or 3200QTrap mass spectrometry system (Sciex) interfaced with an Agilent 1200 HPLC platform. For antimicrobial analysis, compounds were separated on a C18 reversed-phase column (Zorbax Eclipse Plus, 1.8-μm particle size, 50 × 4.6 mm) using a binary gradient. Mobile phase A consisted of 0.2% formic acid in water, while mobile phase B was acetonitrile. The gradient program increased acetonitrile from 5 to 70% over 2 min, held at 70% for 2 min, followed by re-equilibration to initial conditions. For coccidiostats, an isocratic method was employed using a mobile phase comprising 10% aqueous ammonium formate (0.01 M with 0.1% formic acid) and 90% acetonitrile, delivered at 0.5 mL/min. The injection volume was standardized at 5 μL for all analyses. When using the 3200QTrap system, separate injections were required for positive and negative ionization modes, as polarity switching was not utilized.

### GC/MSD in-house analytical method

**Fig. 2:**
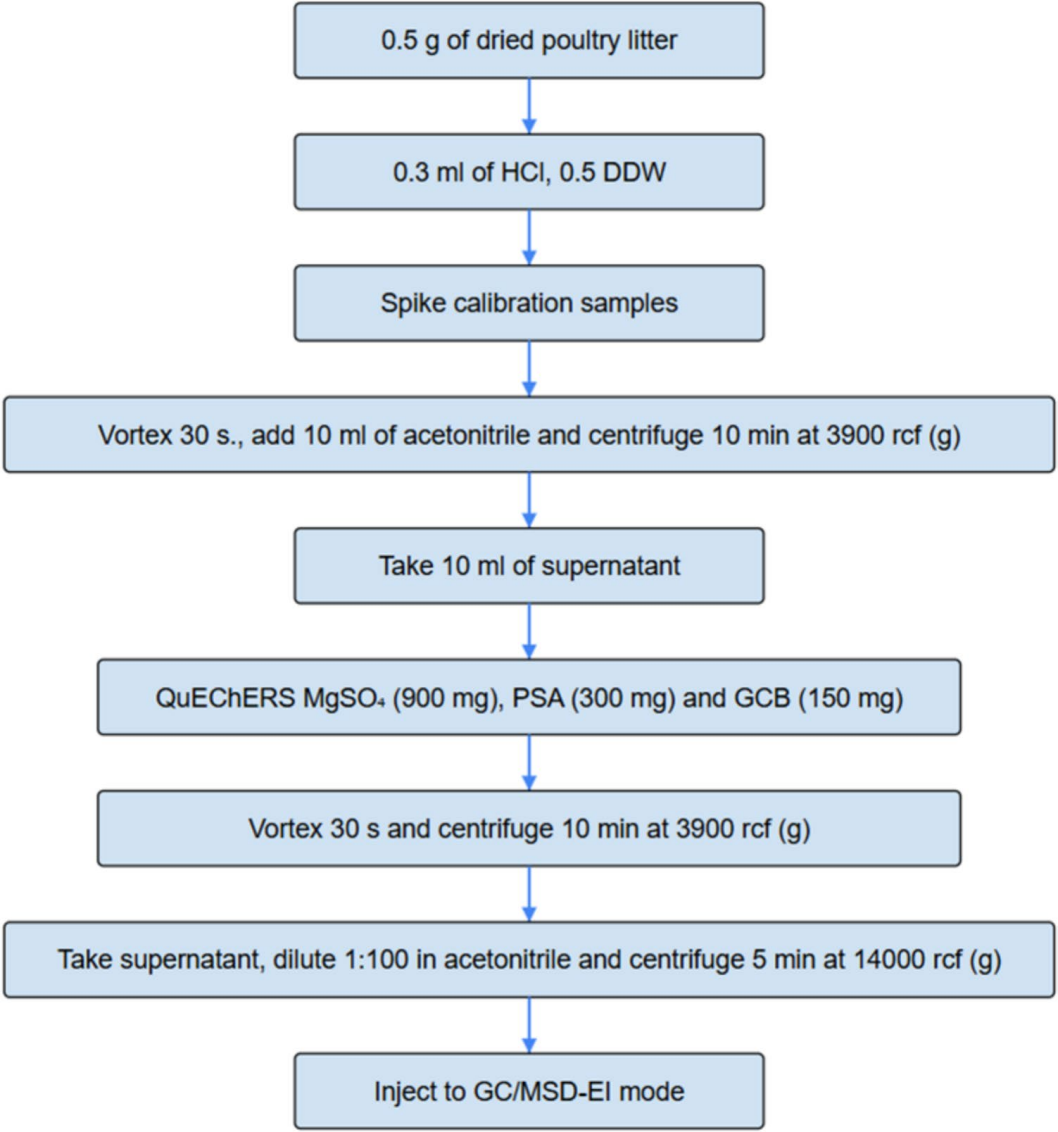
Analytical scheme used for drug SCVFA determination in broiler litter

GC/MSD in-house analytical method were used to detect the SCVFA change during all three treatments (Fig. 2). The split mode of a liner ultra-inert universal low pressure drop and glass wool purchased from Agilent (Munich, Germany) were used in a GC/MSD 5973 apparatus with a DB FATWAX column (Agilent). The gas chromatography (GC) oven temperature were started at 40 °C and raised to 300 °C. The ionization impact mode was set at 70 eV to ionize SCVFAs. MS data were collected in the single ion method (SIM) mode.

### Statistical analysis

Concentrations of anti-microbial and coccidiostat drug residues were examined using Microsoft Excel 2011 and Prism-GraphPad software (version 5.0, San Diego, CA) for statistical evaluation. To determine if there were any significant differences in the mean residue compound levels across various treatments, a one-way analysis of variance (ANOVA) was conducted, followed by a Tukey multiple comparison test. The threshold for statistical significance was established at 0.05.

## Results

### Temperature and moisture reduction by different broiler litter treatments

Broiler poultry litter is a dry waste due to bedding materials, as compared to other feed animal sector producers of manure. Billions of microorganisms can be found per gram of litter. These microbes are responsible for most decomposition and release of heat, water, and carbon dioxide. Microorganisms rely on a suite of enzymes to break down different organic matter in the litter.

**Fig. 3:**
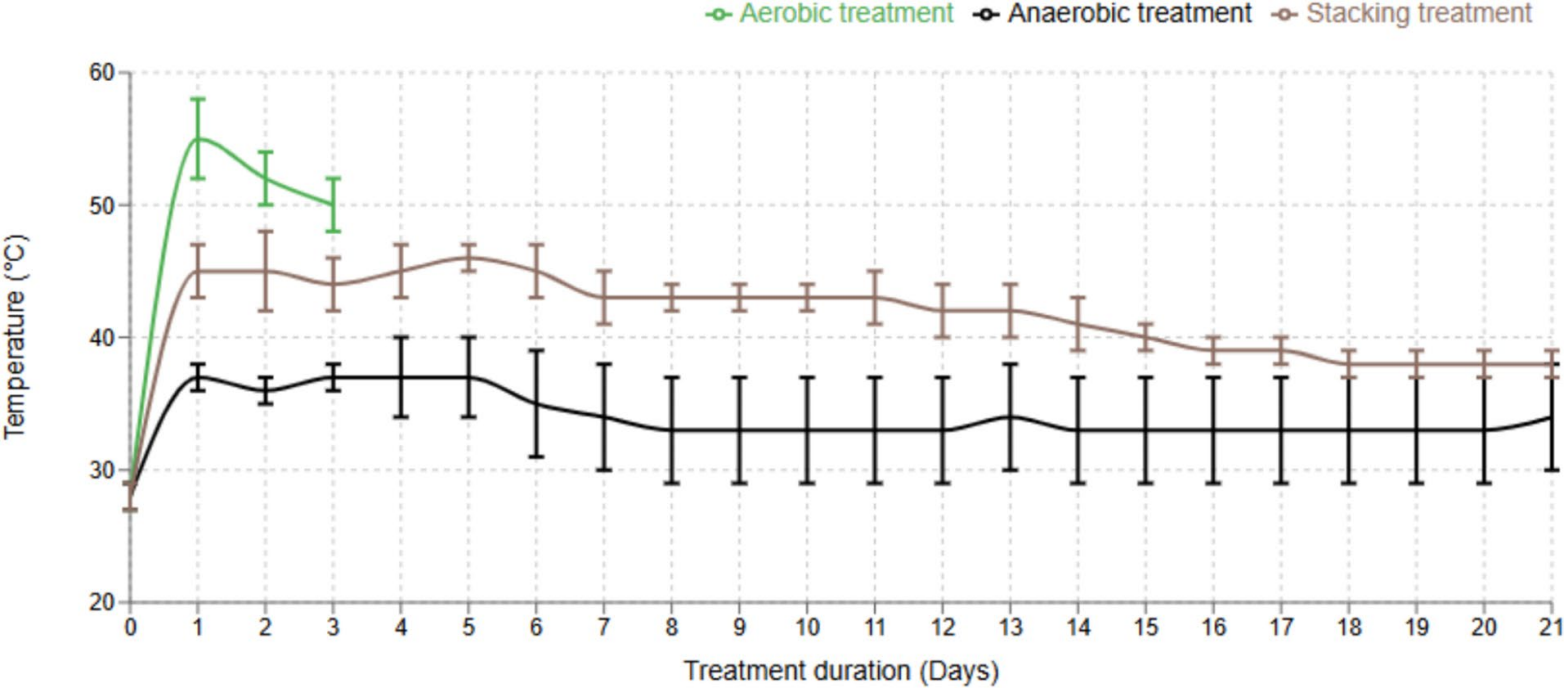
Temperatures in broiler litter during lab-scale treatment (*n* = 4, ± SD)

The peak temperatures in forced aeration litter bins were higher than those in stacking and anaerobic treatments at 60 °C, 50 °C, and 38 °C, respectively (Fig. 3). In aerobic (forced aeration) treatment, the temperature rose rapidly in 24 h and dropped on the second and third days of treatment. In stacking treatment, the temperature rose after 24 h and continued to rise for almost 2 weeks. In anaerobic treatment, temperatures rose from room temperature to 38 °C and remained there (Fig. 3).

### The effect of treatment on pH

**Fig. 4:**
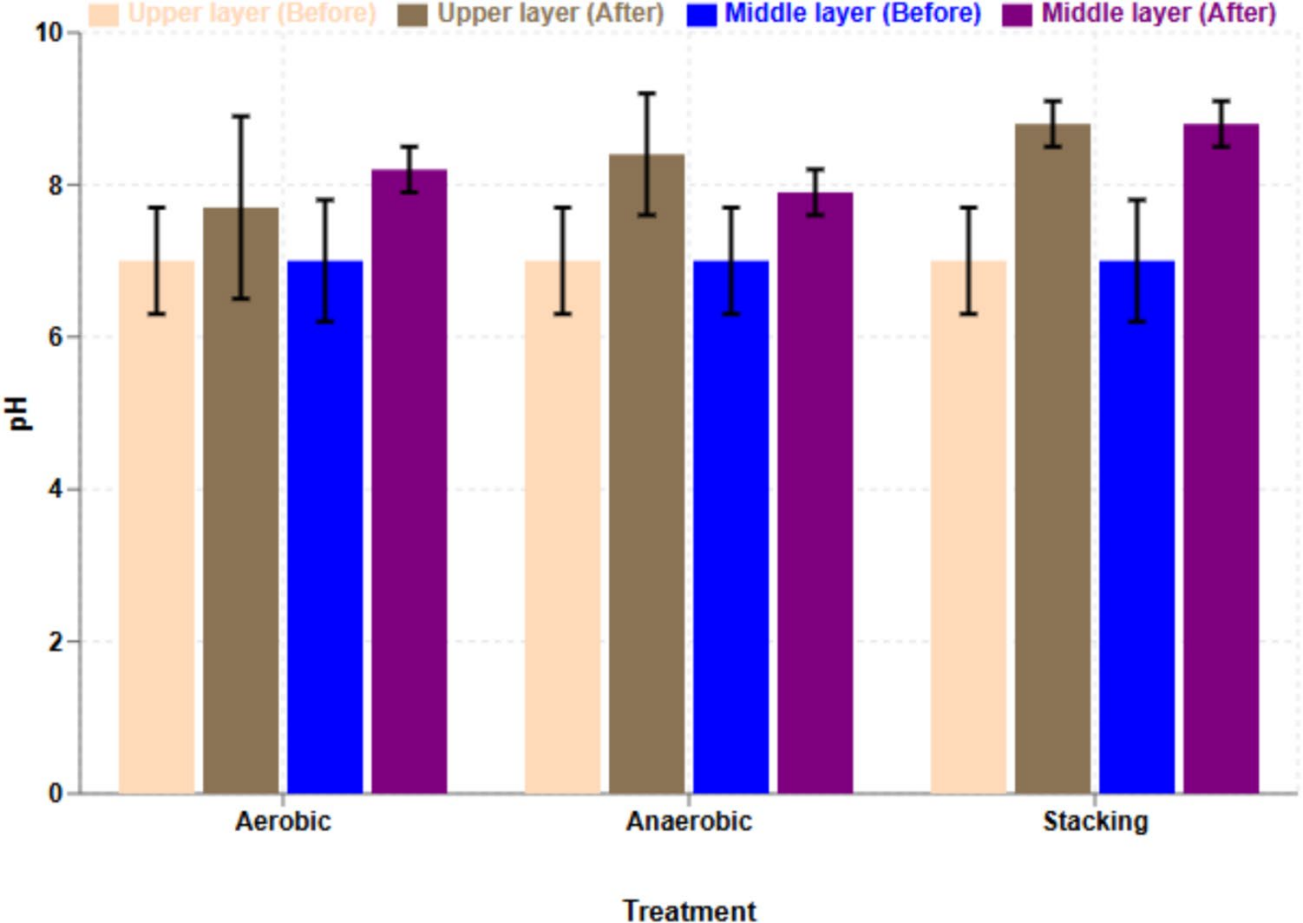
Average poultry litter pH (*n* = 4, ± SD and *p* > 0.05)

The lab-scale investigation of three broiler litter treatments demonstrated significant pH changes throughout the treatment period. Initial pH values prior to treatment averaged 7.2 (± 0.63). Following treatment, measurements from the middle layer revealed distinct pH variations across treatment methods. The anaerobic treatment resulted in a pH of 7.9 (± 0.3), while aerobic treatment yielded 8.2 (± 0.8), and stacking treatment showed the highest pH at 8.8 (± 0.3) (Fig. 4). These findings indicate a consistent trend of pH elevation across all treatment methods, with stacking treatment exhibiting the most pronounced increase from baseline conditions. The pH-dependent behavior of drug residues and their relationship to treatment conditions are further detailed in the supplementary materials (Figure S4), which include comprehensive data on pH effects on various drug residues in aqueous solutions across different temperature ranges.

### The effect of treatment on moisture reduction

**Fig. 5:**
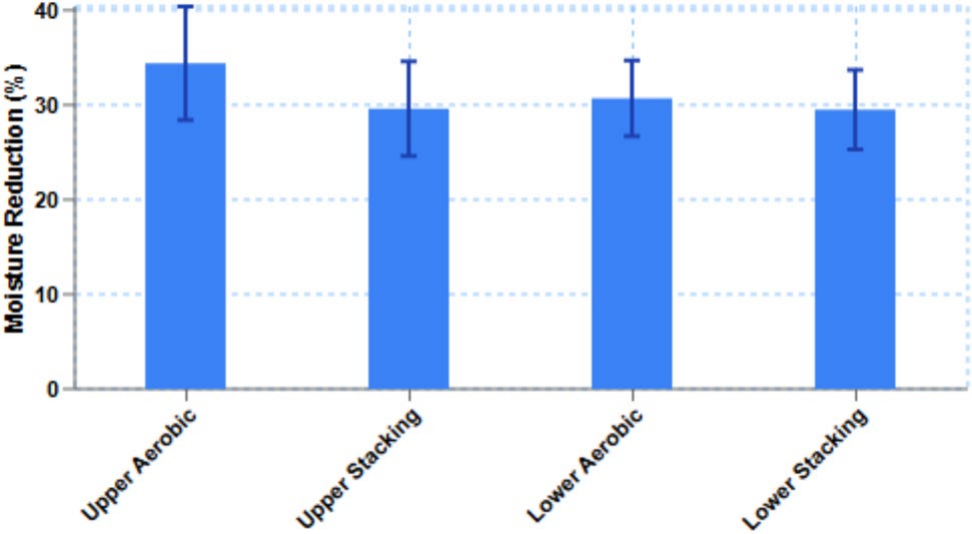
Effect of broiler litter treatment on moisture reduction (*n* = 20, ± SD and *p* > 0.05)

The aerobic and stacking treatments influence moisture content during decomposition of the litter. However, in anaerobic treatment, the reduction of moisture was negligible (Fig. 5).

Moisture reduction in the aerobic and stacking treatment in the lower layer were 30.7% (± 4) and 29.5% (± 4.2), and in the upper layer reductions were 34.4% (± 6) and 29.6% (± 5) after 3 days of treatment in aerobic and 3 weeks in stacking treatment, respectively (Fig. 5).

### The effect of treatment on crude protein content

The main nutrient for ruminant animals in broiler litter is crude protein (non-protein nitrogen and true protein). The average percentages of crude protein in broiler litter after the two treatments were almost the same (32–34.8%).

**Fig. 6:**
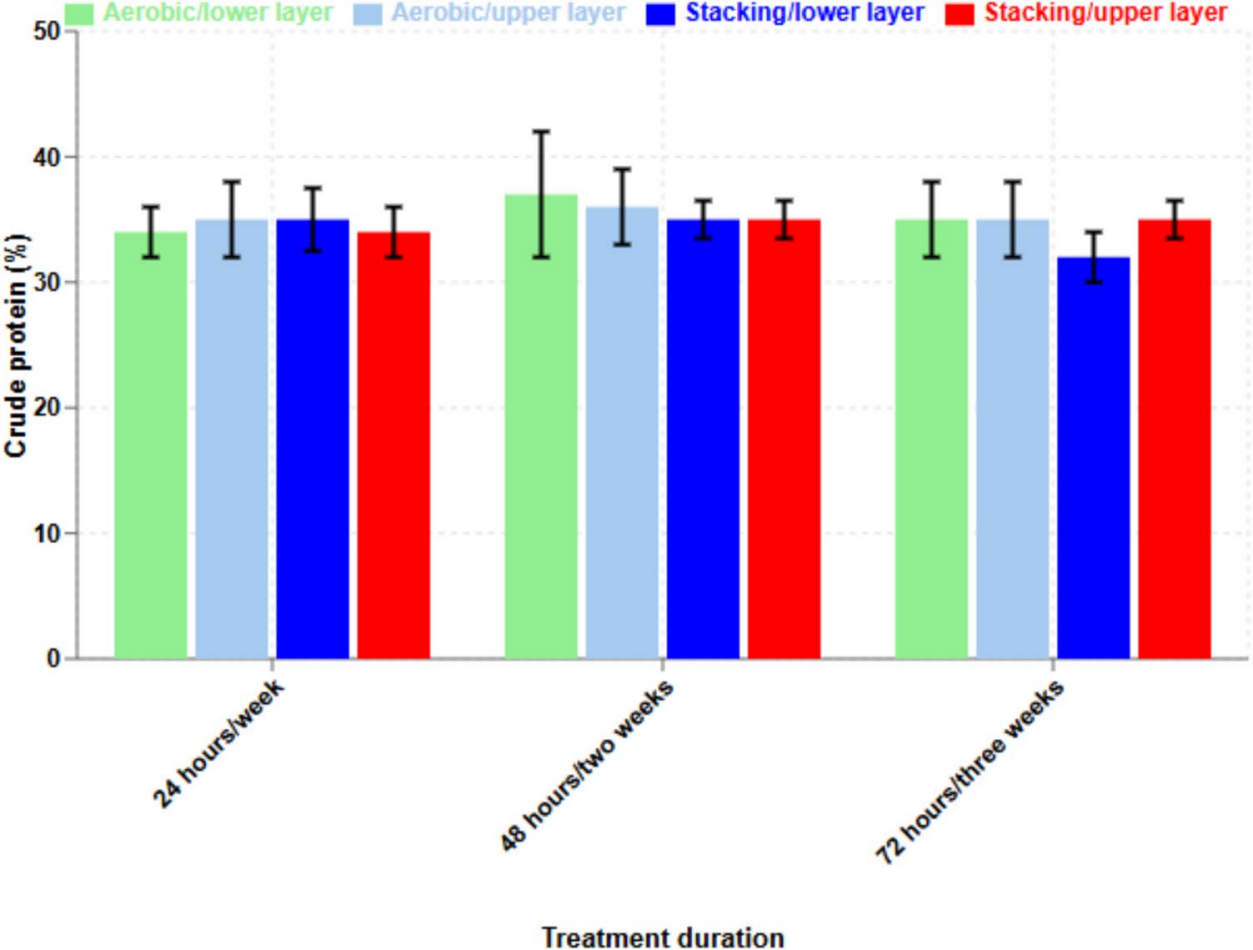
Crude protein percentage in broiler litter (*n* = 9, ± SD and *p* > 0.05)

The crude protein content (± SD) before treatment was 35.5% (± 3). After treatments, the crude protein in the lower layers of aerobic and stacking broiler litter treatments were 34.7% (± 0.9) and 34.8% (± 0.5), respectively, while in the upper layer, values were 34.9% (± 1.2) and 32.5% (± 0.9), respectively (Fig. 6).

### Ash content of broiler litter

Ash content in BL is made up of macro- and micro-minerals from feed, bedding materials, and the excretion of birds and soil.

**Fig. 7:**
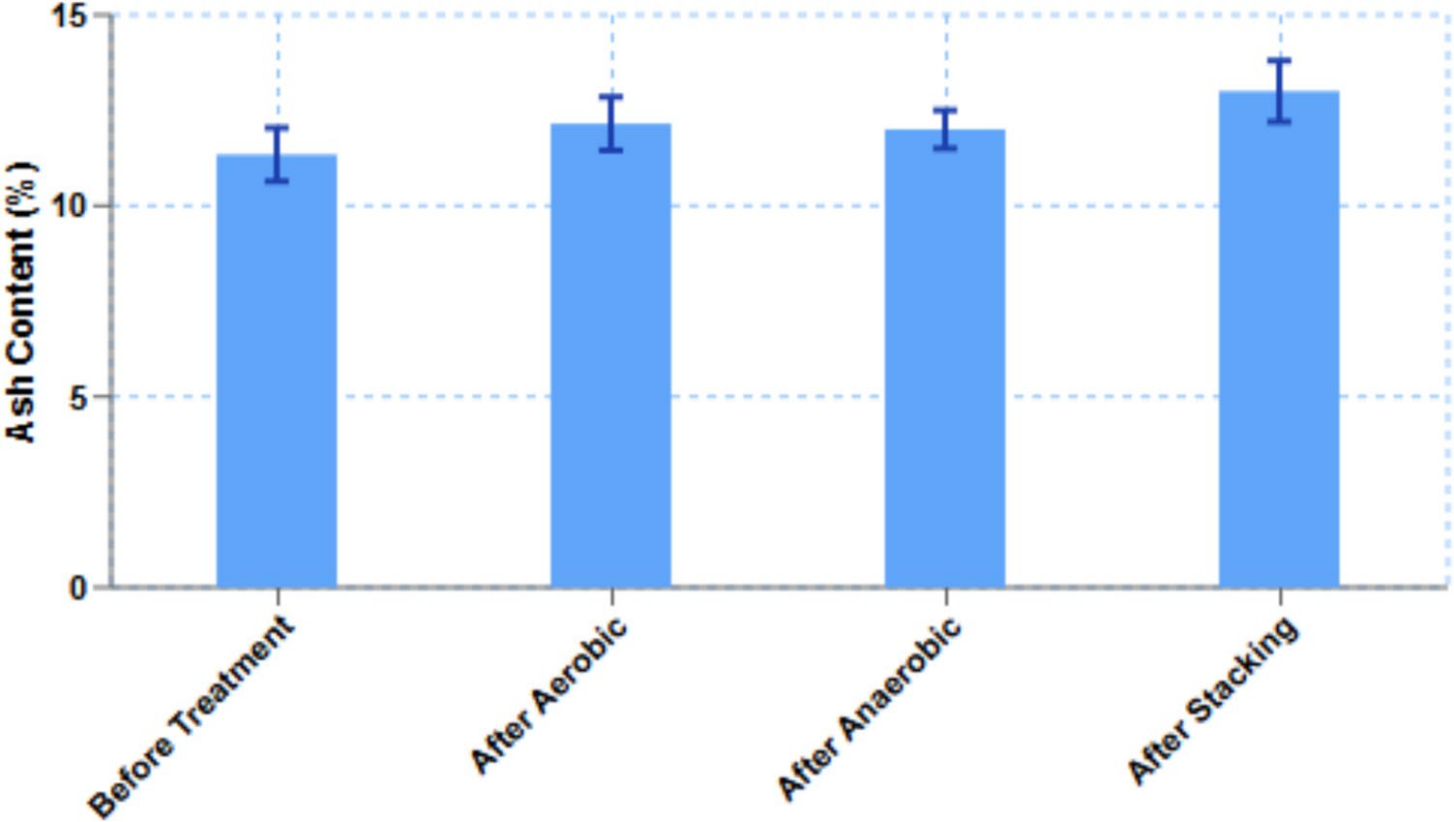
Ash content in broiler litter (*n* = 4, ± SD and *p* > 0.05)

The percentage (± SD) of ash content before BL treatment were 11.34% (± 0.7). After aerobic, anaerobic and stacking treatment were 12.15% (± 0.7), 12.0% (± 0.5), and 13% (± 0.8), respectively (Fig. 7).

### SCVFA in broiler litter

Acetic, propionic, valeric, hexanoic, and isovaleric acids were detected in the electron impact (EI) mode of the ion source in GC/MSD. The retention times of the corresponding peaks were 8.042, 8.828, 9.930, 10.485, and 11.371 min, respectively. SCVFA percentages after anaerobic treatment are indicative of fermentation by a treatment system. However, during a 21-day study, BL without any other carbon source showed low levels of the fermentation product of acetic, propionic, valeric, hexanoic, and isovaleric acids, as shown in Figure S5. In anaerobic lab-scale BL treatments, SCFA concentrations did not increase (Figure S5), although there was a bad odors and pH > 6 in the lab-scale experiment.

### Degradation of antimicrobial and cocccidiostats

**Fig. 8:**
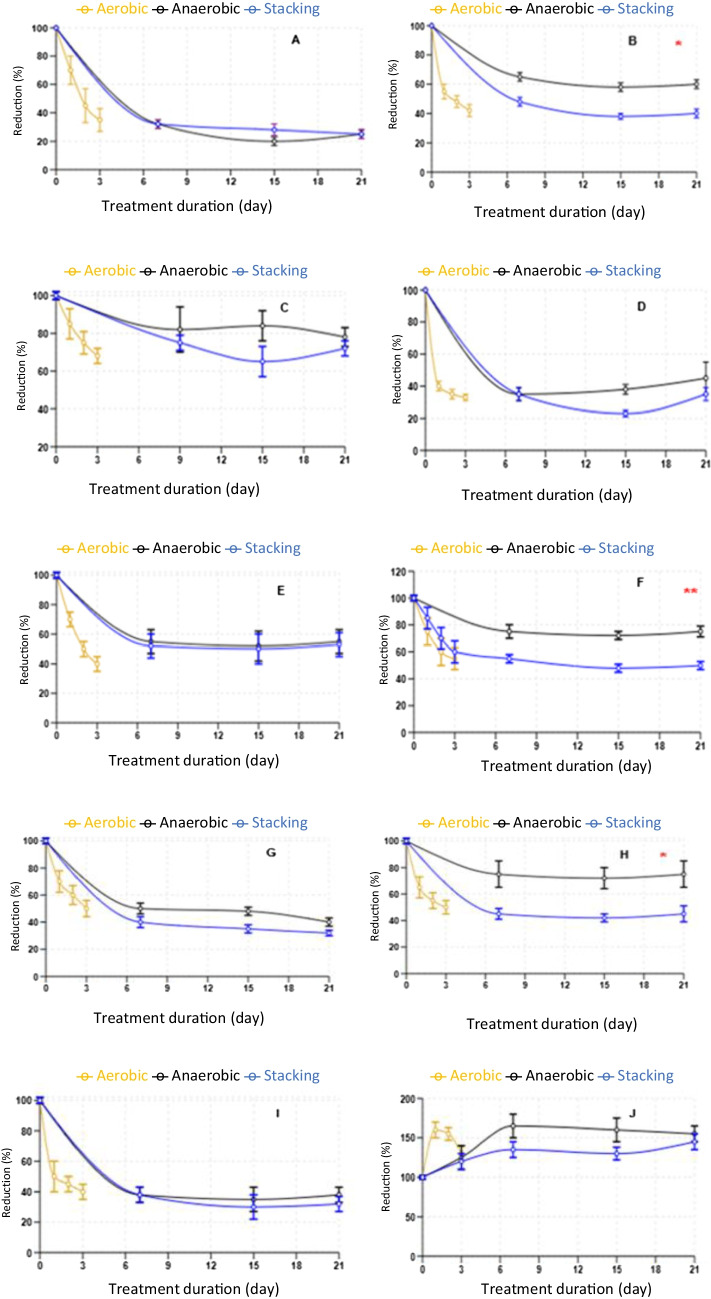
Residual percentages of ten antimicrobial and coccidiostat drugs after aerobic, anaerobic, and stacking poultry litter treatment. Residue percentages (± SEM) during 72 h and 3 weeks of treatment at low spiked concentration of 0.5, 1, 1.5, and 2 ppm of tilmicosin (**B**), maduramicin (**C**), clopidol (**D**), narasin (**E**), monensin (**F**), robenidien (**G**), diclazuril (**H**), nicarbazin (**I**), and dicoquinat (**J**). *n* = 20, 20, 20, 18, 15, 17, 18, 15, and 20, respectively. (*p < 0.05, anaerobic and stacking). (**p < 0.05, aerobic and stacking)

Lab-scale experiments examining the breakdown of spiked antimicrobial and coccidiostat samples revealed that five antimicrobial categories—tetracycline, fluoroquinolones, macrolides, sulphonamides, and beta-lactams—exhibited degradation rates exceeding 95% at low residue levels (Table S1). This group included tetracycline, doxycycline, oxyteteracycline, chlortertracycline, amoxicillin, ciprofloxacin, danofloxacin, enrofloxacin, norfloxacin, sulfisoxazole, sulfachloropyrazine, sulfachloropyridazine, sulfadiazine, sulfadimidine, sulfadoxine, sulfadimethoxine, tylosine, and erythromycin. The sole exception was tilmicosin, which showed a 65% degradation rate (Fig. 8A). These results were observed during 72-h aerobic treatments and 3-week stacking and anaerobic lab-scale procedures. With the exception of salinomycin, all eight coccidiostats remained present in BL (Fig. 8C–J), exhibiting varying degrees of degradation. For five representative antimicrobial drugs—sulfisoxazole, amoxicillin, erythromycin, tetracycline, and ciprofloxacin—at elevated concentrations of 5, 10, 15, and 20 ppm, the degradation rate exceeded 95% across all three treatment methods, with erythromycin being the sole exception (Fig. 8A, *n* = 20).

The study results indicated substantial degradation rates during a 3-day aerobic treatment and 7-day anaerobic and stacking treatments. At low spiked concentrations of 0.5–2 ppm, tilmicosin showed greater persistence compared to macrolide groups (erythromycin and tylosin). The residue percentages (± SEM) for tilmicosin were 35 (± 2.6), 56 (± 1.3), and 43 (± 1.7) for stacking, anaerobic, and aerobic treatments, respectively. For clopidol, these values were 36.4 (± 3.2), 42.8 (± 10.9), and 33 (± 2.5), respectively. Both tilmicosin and clopidol are infrequently used on Israeli broiler farms. The four coccidiostats used in Israel at low frequencies are decoquinat, diclaziuril, maduramycin, and robendien. Following stacking treatment, their residue percentages were > 100 (± 9.9), 43.7 (± 5.7), 73, and 32.7 (± 3) %, respectively. After aerobic treatment, the values were > 100 (± 9.2), 51.4 (± 2.5), 67.5 (± 2.2), and 48.4 (± 5), respectively. Three weeks of anaerobic treatment resulted in values of > 100 (± 13.4), 74.7 (± 7.7), 80.5 (± 5), and 38.1 (± 2.6) for similarly spiked decoquinat, diclaziuril, maduramycin, and robendien. For the most commonly used coccidiostats—narasin, monensin, and nicarbazine—degradation percentages were 54.9 (± 5.4), 49 (± 2.1), and 36.5 (± 3) for stacking treatment; 53.3 (± 3.8), 74 (± 5.4), and 32.2 (± 3.6) for anaerobic treatment; and 42.7 (± 4.6), 59 (± 6.4), and 40 (± 2.7) for aerobic treatment, respectively. Figure 8 illustrates the degradation percentages of ten antimicrobial and coccidiostat drugs following stacking broiler treatment.

## Discussion

In investigating the effects of different BL treatments, we saw that pile size, porosity, moisture content, and climate changes influenced temperature increases in aerobic and stacking treatments. The most important parameters in decomposing litter were temperature, oxygen, and moisture in the litter. In our studies, the highest temperature occurred in aerobic (active aeration) treatment for 72 h was almost 55–65 °C. Composting treatments also reach these temperatures, aligning with the US FDA’s food safety and modernization act (FSMA) guidelines (FDA 2016) for agricultural applications. These guidelines recommend such temperatures due to their effectiveness in eliminating most pathogenic bacteria that pose risks to environmental and human health. Supplying air during treatment is an essential factor for microorganism activity and release of water and carbon dioxide from the litter. A fast reduction of temperature occurred in aerobic treatment due to the size of the treatment sample. In the case of previously reported manure-composting treatment, the effect of sample size on treatment was also described, as high temperatures > 40 °C were maintained longer in large windrows than in small windrows (Tirado and Michel 2010; Hu et al. 2009). In some studies, it was shown that the duration of the temperature rise was not dependent on presence of antimicrobials in static and turning swine manure and poultry litter (Bao et al. 2009; Hu et al. 2011). However, when we spiked erythromycin at low and high concentrations, the degradation percentages were significantly different. The most degradation was seen on the first day of aerobic treatment and after 1 week with the other treatments. Comparing the effect of treatments on degradation per day revealed that the most degradation had occurred upon aerobic treatment. This shows that oxygen availability is important for reducing treatment duration and the degradation of drug residues. The importance of oxygen availability for antimicrobial degradation was previously discussed (Ali et al. 2013). Supplying oxygen raised the temperature, and increasing microorganismal degradation of drug residues and decomposition was more effective than what was seen with non-oxygen-supplying treatment.

In lab-scale anaerobic BL treatments, the SCVFA concentration did not increase and a bad odors were noticed with high pH values (> 6). This might be attributed to the low content of carbon which increased the buffering capacity of uric acid and might cause reduced fermentation in anaerobic treatment (Acharya 1935; Jungbluth et al. 2001; Chadwick 2005).

Crude protein contents in BL were not significantly different in the middle and upper layers before and after treatment (aerobic and stacking). Total nitrogen concentrations during lab-scale treatment were not changed. However, some studies reported reduction of total nitrogen during composting (Dolliver et al. 2008; Ramaswamy et al. 2010). Contents of crude protein and ash in BL were significantly different than those measured in 1970 (18 and 24%, respectively, Figure S6, https://akol.co.il/icbaapp/articles/0131/0131.1974.04.pdf). The main differences were due to the old conventional housing system and the modern controlled farming system on moisture content of BL. In the modern controlled farming system, moisture content is controlled and the release of non-protein nitrogen (NPN) in BL litter is reduced. The effects on moisture reduction by the stacking treatment during 3 weeks and the aerobic treatment for 3 days were between 29.5 and 34.6%.

The findings from our stacking approach exhibited comparable outcomes to those reported in studies examining the impact of composting on the breakdown of certain pharmaceutical residues, including tetracycline, fluoroquinolone, and various coccidiostats (Esperon et al. 2020; Subirats et al. 2020). The chlortetracycline degradation ratio remained low after a week of turkey litter composting, mirroring our findings. However, significant variations were observed in the breakdown rates of mononsin, tylosin, and sulfamethazine (54%, 76%, and 0%, respectively) in the turkey composting experiment, contrasting with our BL degradation studies using three distinct methods. The minimal degradation of erythromycin following a high-concentration addition may be attributed to reduced Pseudomonas bacterial activity in the litter. A prior investigation demonstrated that *P. aerugiona* was among the bacteria capable of biodegrading erythromycin compounds (Sabic et al. 2015).

The degradation of antimicrobials and coccidiostats is a function of bacterial activity, self-heating, and self-degradation. The degradation rate of the tested drugs, relative to the treatment, was stacking > aerobic > anaerobic for tilmicosin, and diclazuril. The current study revealed notable variations among these compounds within treatment (*P* < 0.05). According to Sun et al. (2014), the biodegradation of ionophores is influenced by temperature and moisture conditions. Our findings demonstrated that increased temperature combined with oxygen supplementation resulted in a higher degradation rate compared to anaerobic treatment methods. Interestingly, a study utilizing a piling and turning composting system for salinomycin and narasin degradation showed contrasting results (Munaretto et al. 2016). Sun et al. (2014) also noted similarities in the degradation of narasin and a synthetic version of narasin containing a methyl group (salinomycin), as well as their degradation ratios (Sun et al. 2022). However, in our study, salinomycin degradation exceeded 95%, while narasin degradation ranged from 46 to 58% across the three different treatment systems. Natural antimicrobials and coccidiostats were more biodegradable, as compared to synthetic compounds and log kow > 2 during all three treatments. Further study should be done on the decoquinate phenomenon concentration that was increased during all three treatments. It can be speculated that decoquinate is a stable molecule and resisted physical and biological degradation processes. The matrix effect for this compound was thoroughly evaluated in our previous work (Efriem et al. 2024), showing minimal interference at 5.1%. Method validation demonstrated robust performance with intra- and inter-day accuracies of 105% and 94% respectively at LOQ. While we investigated potential conversion from quinolone derivatives, our findings indicated that the elevated decoquinate concentrations were not attributable to quinolone transformation; however, this unexpected increase in decoquinate concentration warrants further investigation to fully understand the underlying mechanisms and potential interactions in complex broiler litter matrices.

Our analysis revealed no significant correlation between different spiking concentrations and degradation patterns, as shown in Figure S7, which may be attributed to the relatively small sample size (200 mg) introduced into the treatment pile; this finding aligns with Bao et al. (2009) and Hu et al. (2011), who demonstrated that temperature patterns in manure treatment were independent of antimicrobial presence; the observed residue reduction appears driven by both physicochemical processes and microbial decomposition, rather than residue drug concentration-dependent interactions.

Environmental and health risks require careful evaluation of degradation efficiency and residual concentrations in treated broiler litter, even with high degradation (> 95%); residual concentrations must be compared to antibiotic resistance thresholds and risk thresholds of ionophores toxicity to ruminants including the following: Narasin at 100–200 mg/kg (Alexander et al. 2007), Maduramicin at < 4.8 mg/kg (Shlosberg et al. 1997), and Monensin at < 200 mg/kg (Gonzalez et al. 2005). These toxicological values emphasize the importance of achieving optimal degradation to maintain safety margins well below these toxicity thresholds; therefore, continuous monitoring and treatment optimization is essential to ensure residual concentrations stay below critical levels when using treated broiler litter for soil amendment or ruminant feed.

## Conclusions

The extensive application of human antibiotics in livestock operations has significantly contributed to the emergence of antibiotic-resistant bacteria, posing risks to both human health and environmental ecosystems. Our research presents the first comprehensive comparison of three economically viable treatment methods for broiler litter (BL), demonstrating that temperature, moisture content, windrow size, and oxygen availability are critical parameters influencing the degradation of pharmaceutical residues and treatment duration in aerobic and stacking systems. The stacking treatment over 3 weeks and aerobic treatment over 3 days effectively reduced moisture content by 29.5–34.6%, significantly improving storage properties and facilitating integration into ruminant feed formulations. Notably, our findings revealed that these biotic treatments achieved degradation rates exceeding 95% for most antimicrobial compounds while maintaining consistent crude protein levels of 32–34.8%, making treated BL an economically attractive protein source for ruminant nutrition. The research provides innovative insights into optimizing BL treatment processes, establishing a framework for implementing cost-effective methods that simultaneously address environmental concerns, antimicrobial resistance risks, and nutritional requirements in livestock production systems. Through systematic evaluation of degradation patterns for 29 pharmaceutical compounds, our study offers practical guidelines for agricultural operations to effectively incorporate treated BL into animal feed while minimizing residual drug content. This work represents a significant advancement in understanding the relationship between treatment parameters and pharmaceutical degradation, contributing to the development of more sustainable and environmentally responsible livestock management practices.

## Supplementary Information

Below is the link to the electronic supplementary material.

Supplementary file1 (DOCX 3150 KB)

## Data Availability

All data will be available on request to the corresponding author.
