# Supplementary material for: The effects of biotic treatments on degradation of antimicrobials and coccidiostats in broiler litter used as ruminant feed
Source: Environ Sci Pollut Res Int. 2025 May 26;32(24):14537–49. doi: 10.1007/s11356-025-36535-9 (PMC12202678; doi:10.1007/s11356-025-36535-9)
Supplement: Supplementary file 1 — Supplementary file1 (DOCX 3150 KB) [file 11356_2025_36535_MOESM1_ESM.docx]

**Supplementary Information for** **Environmental Science and Pollution Journal**


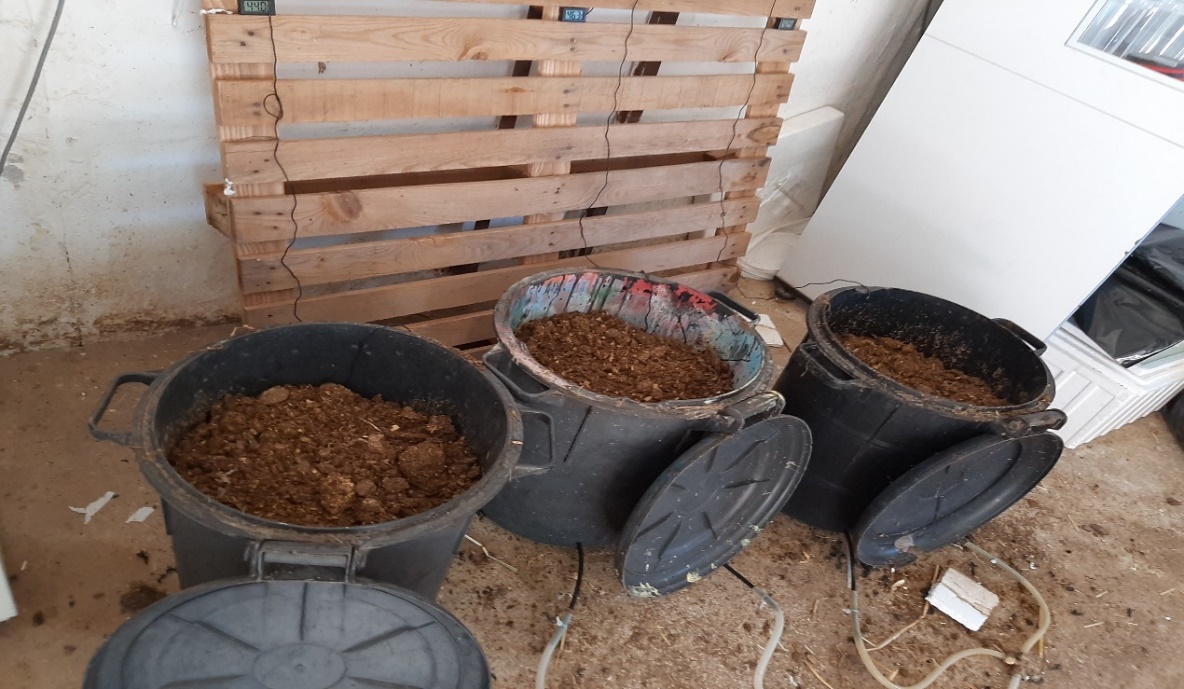


72 hours

48 hours

24 hours

**Figure S1**. Three bottom drill containers for active aerobic treatment in a lab-scale study


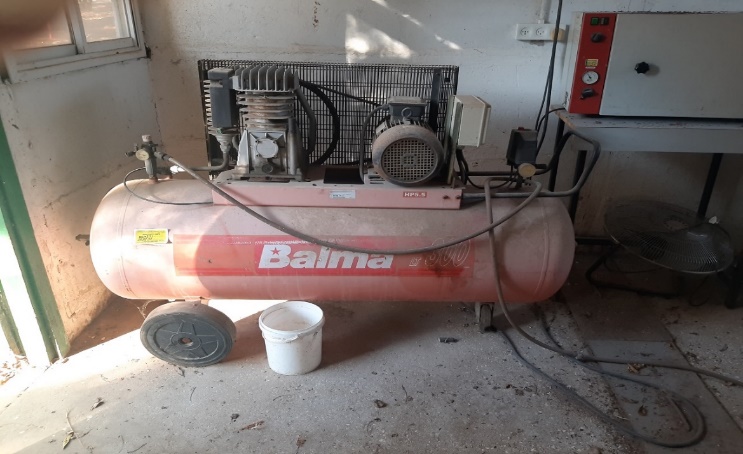

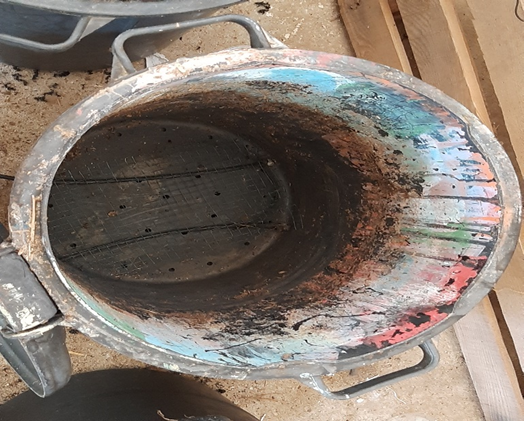


Compressor

Drill jar and oxygen supply tube

Drill jar

**Figure S2.** A compressor for active aerobic treatment in a lab-scale study.


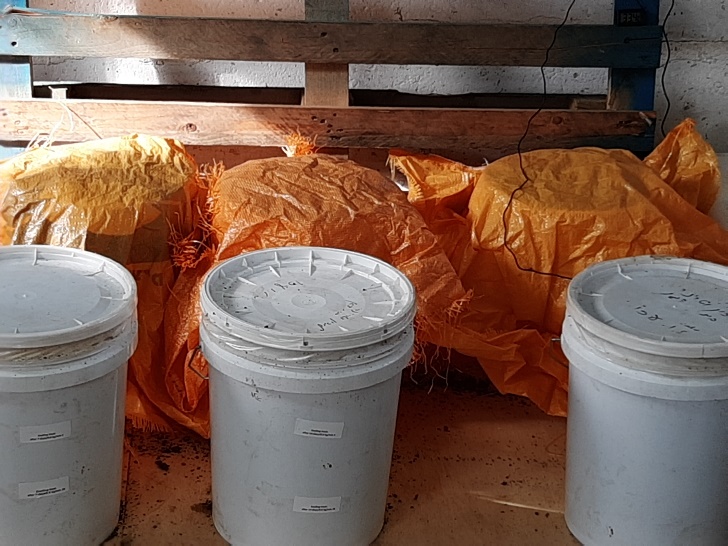


**Figure S3.** Stacking and anaerobic treatment plastic bins


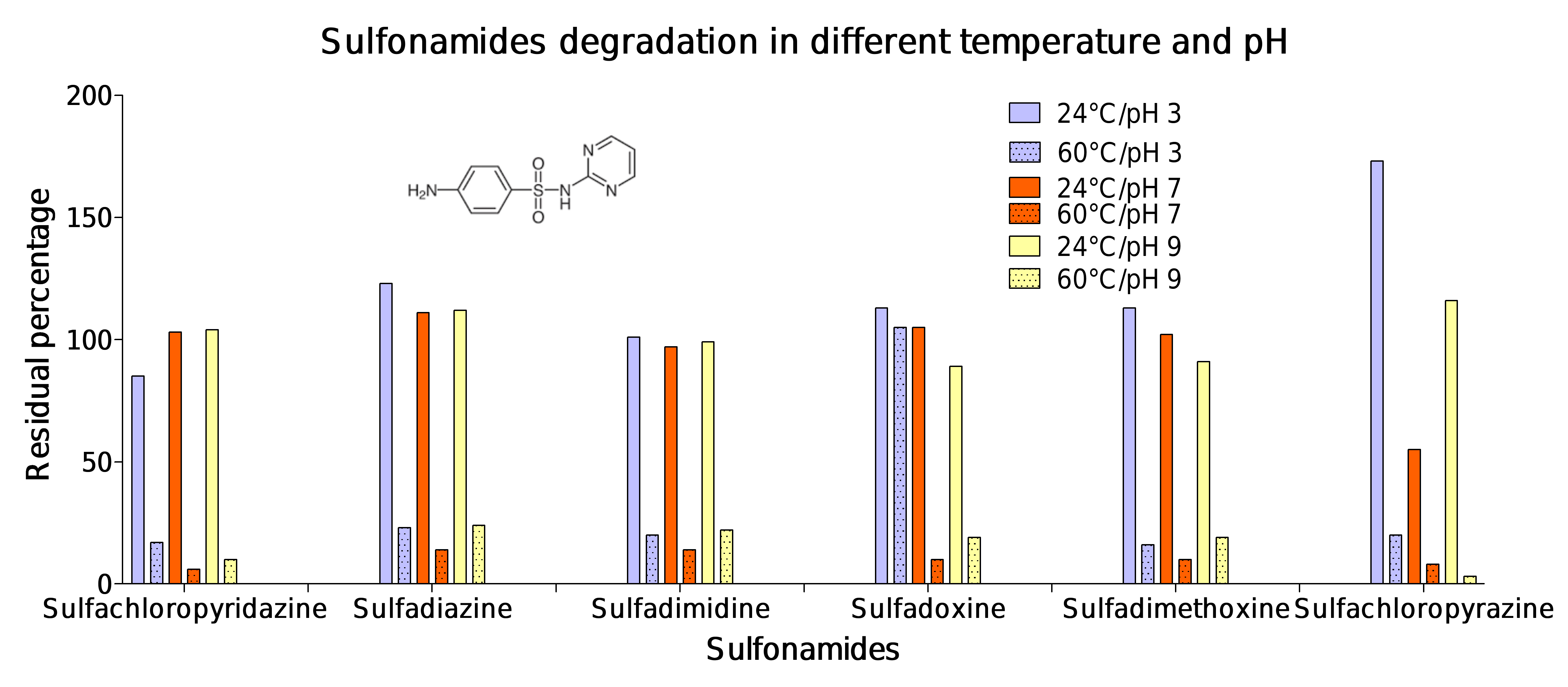

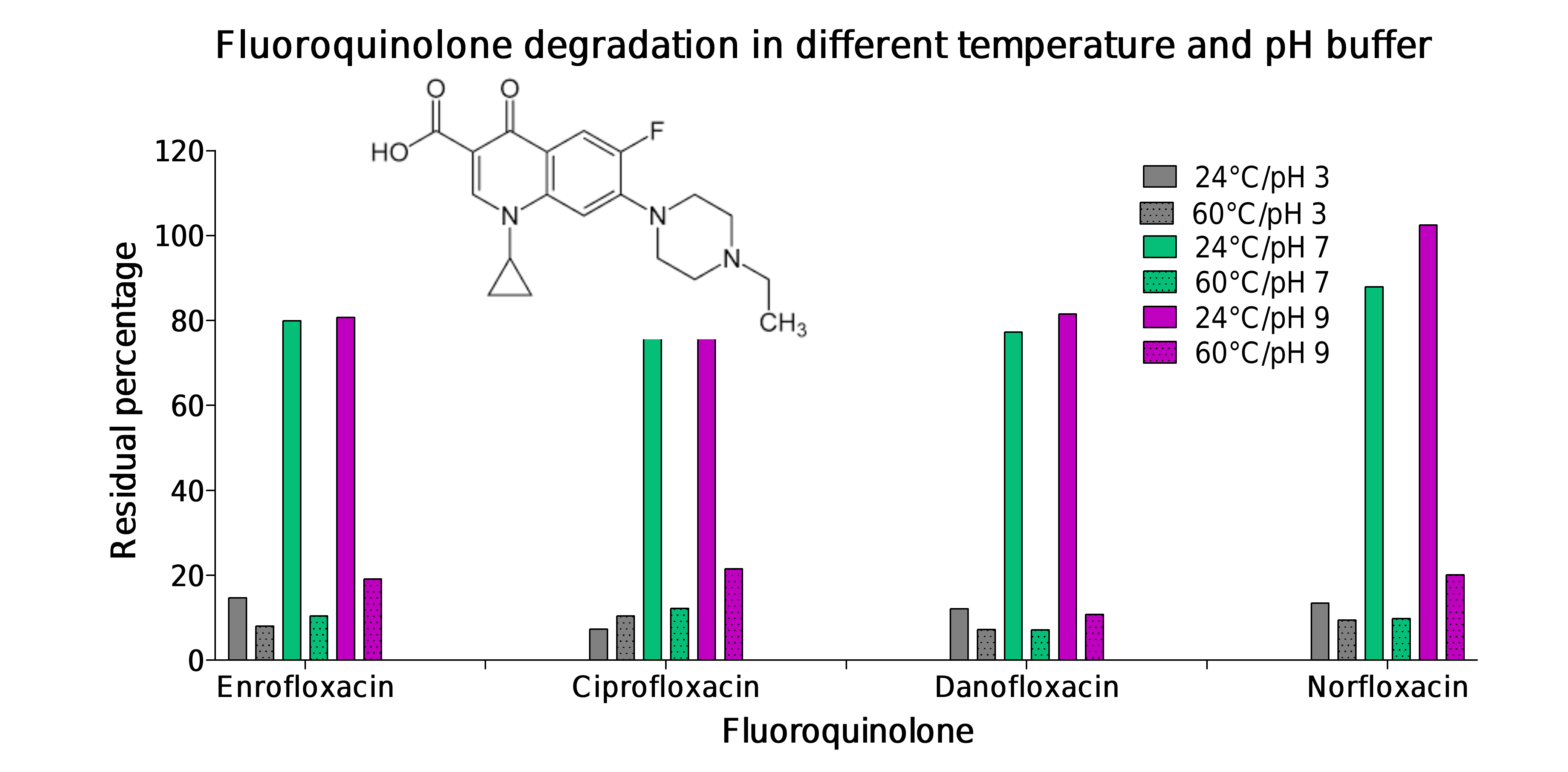


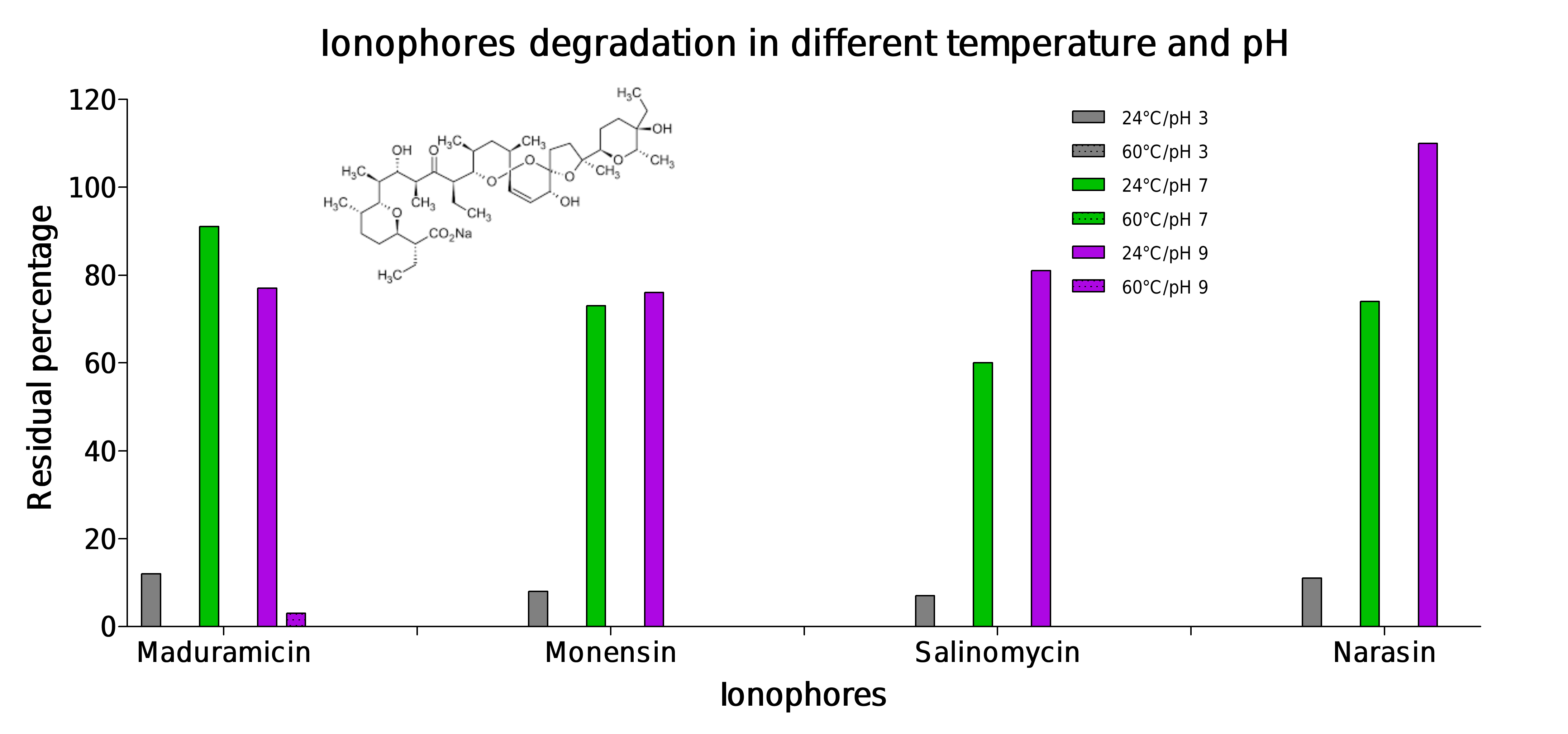

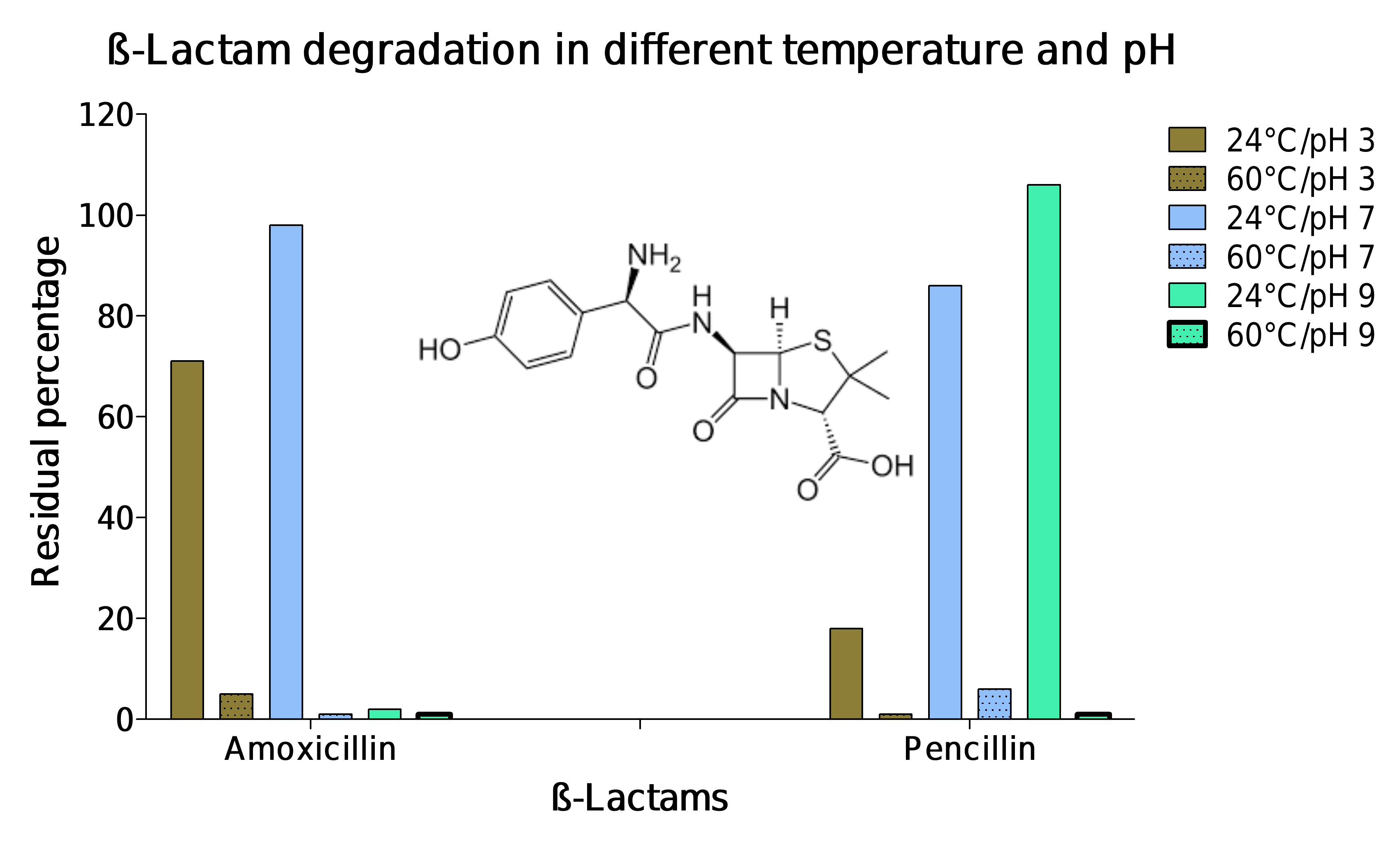


**
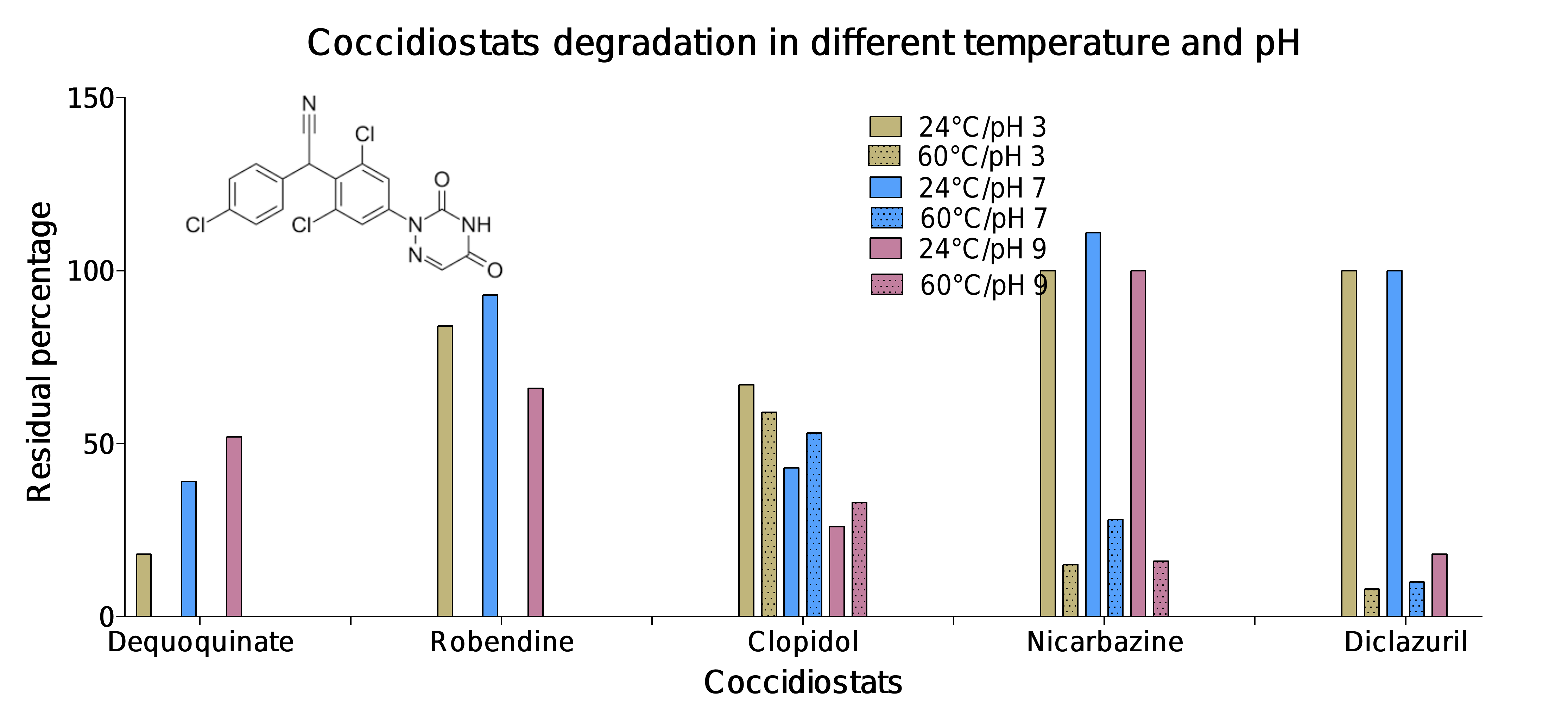
**

**Figure S4.** The effects of pH and temperature on the stability of anti-microbials and coccidiostats in double-distilled water (n=3).

**Figure S5.** Relative concentrations of isovaleric, valeric, acetic, hexanoic and propionic acid before and after broiler litter treatment.


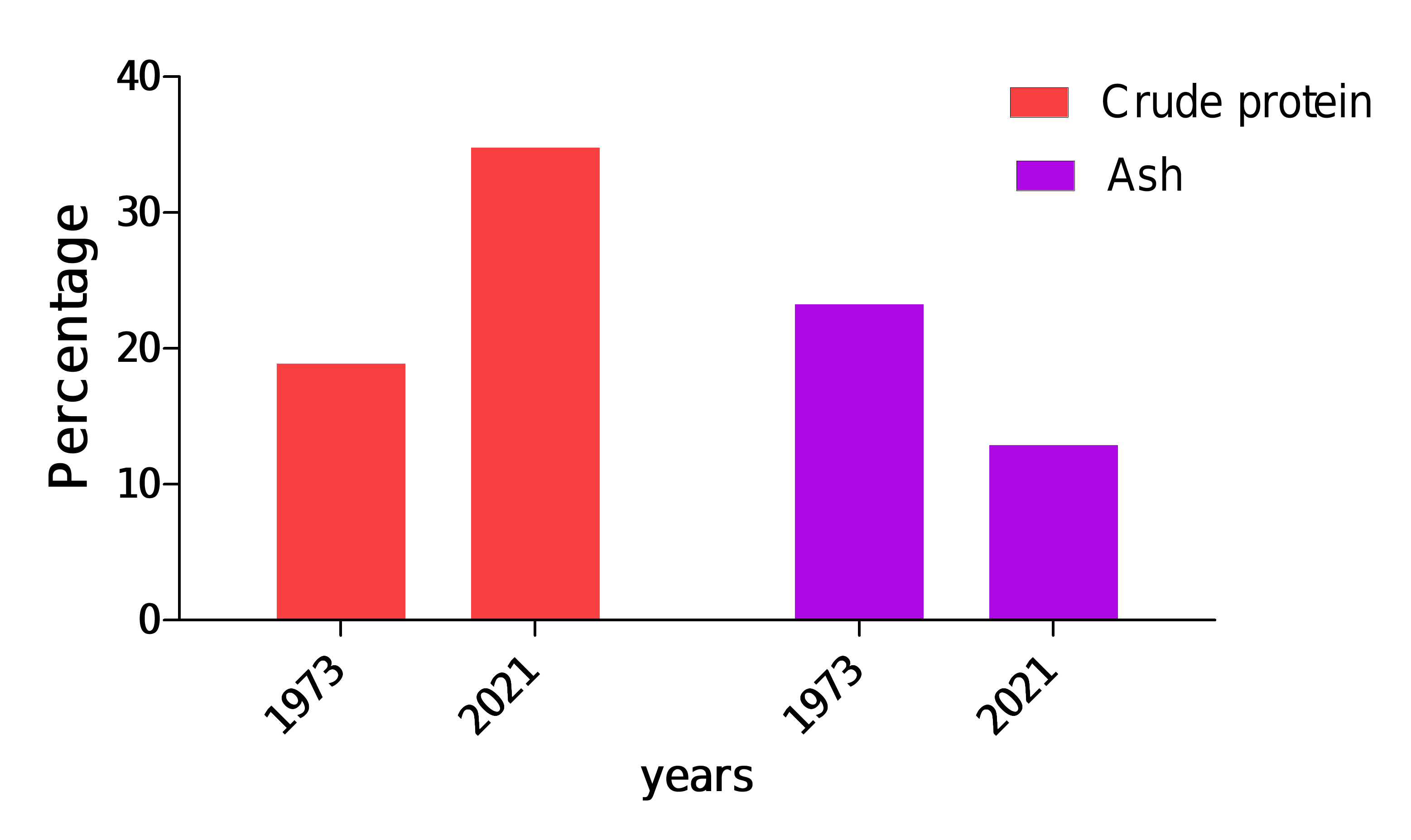


**Figure S6.** Crude protein and ash content in broiler litter in 1973 (n=3) and 2021. Source: <https://akol.co.il/icbaapp/articles/0131/0131.1974.04.pdf>


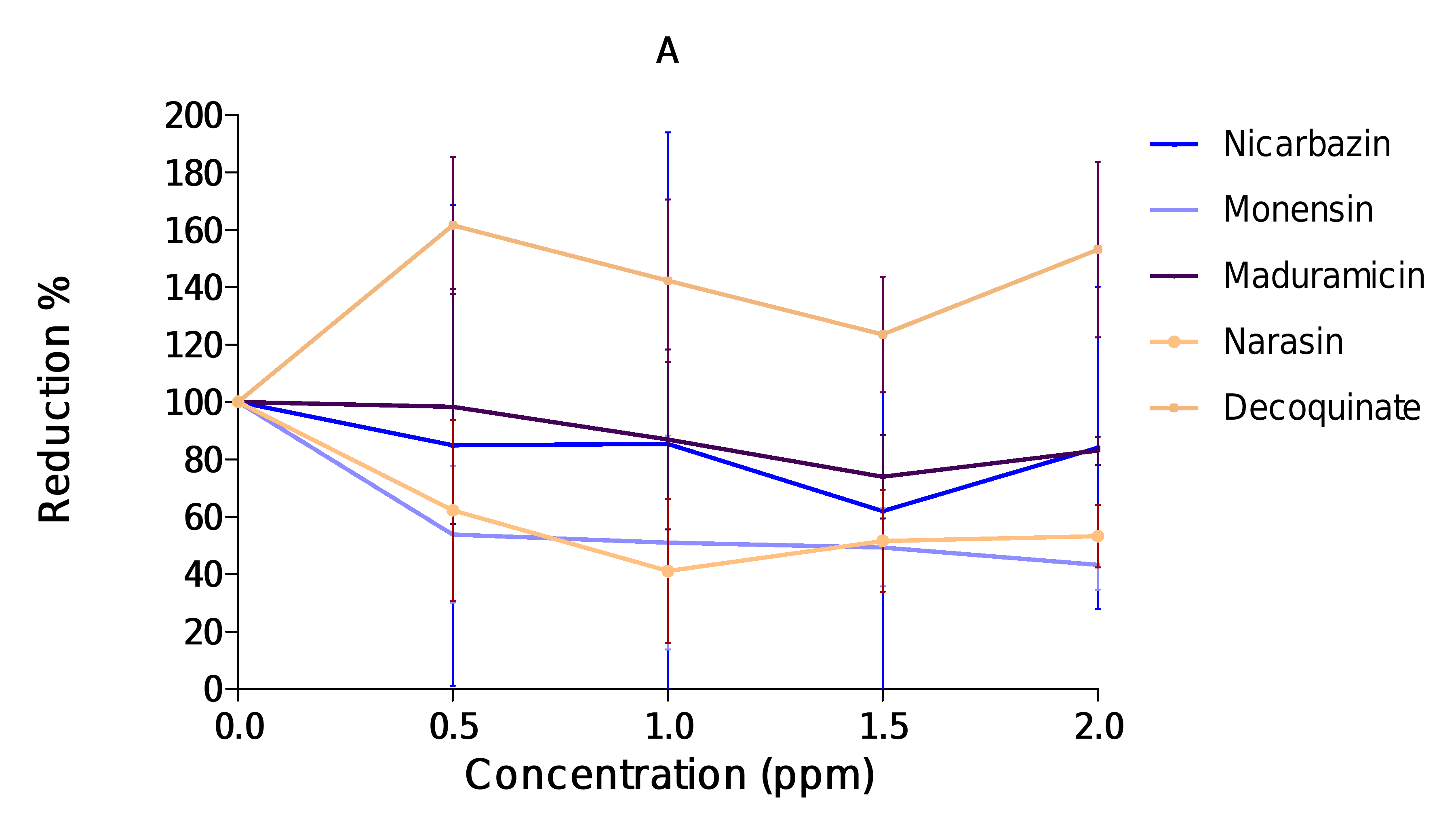

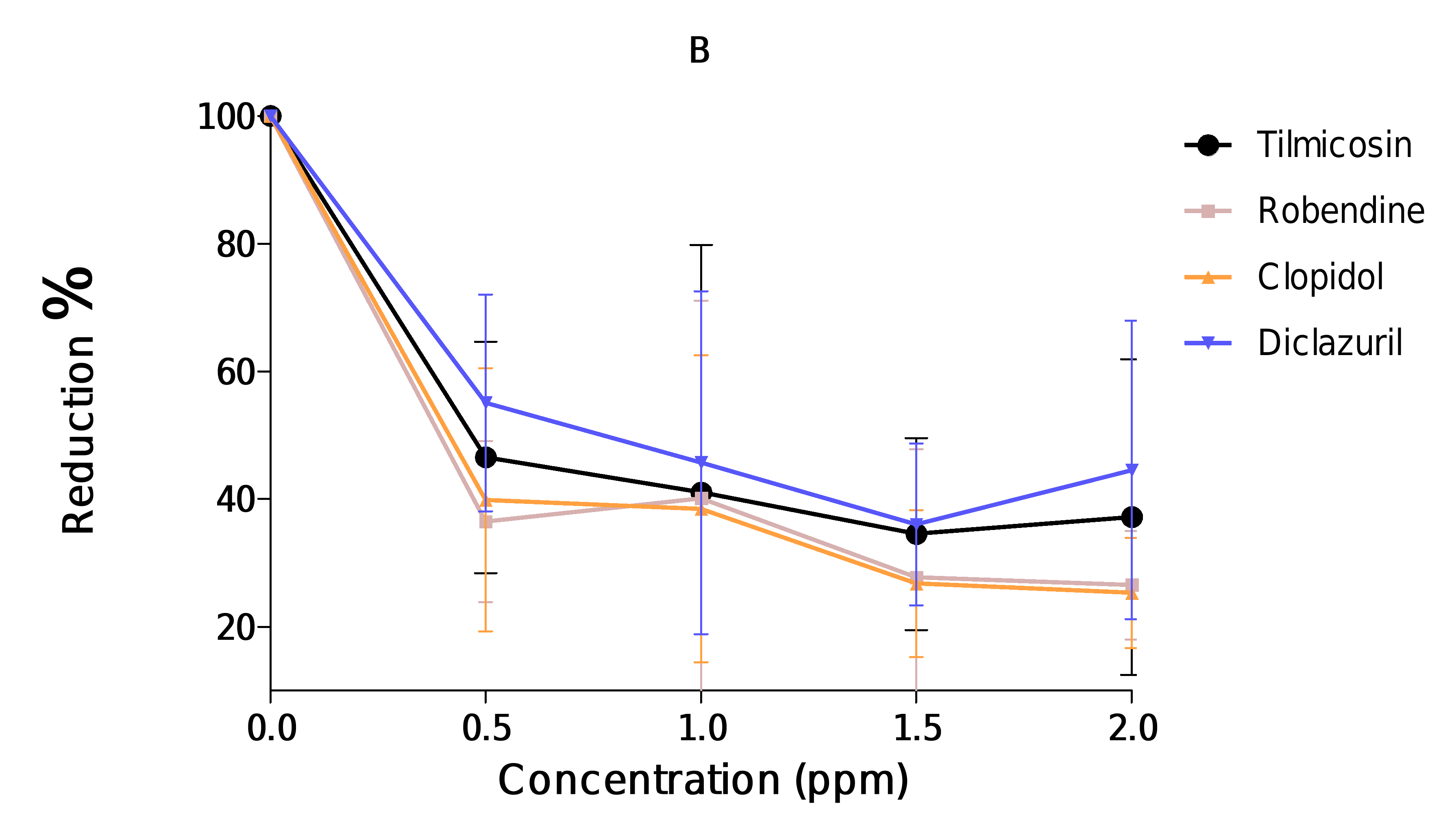


**Figure S7.** Relative concentrations of ten anti-microbial and coccidiostats upon stacking BL treatment after 21 days and at different initial concentrations of 0.5, 1, 1.5 and 2 ppm (mg/L, n=3).

**Table S1**. Antimicrobial and coccidiostat drug residue percentages after various lab scale treatment,spike concentration at 2 ppm

| Group | Compound | conc x№ of batches | Reduction % after treatment |  |  |
| --- | --- | --- | --- | --- | --- |
|  |  | (n) | Aerobic | Anaerobic | Stacking |
| Tetracyclines | tetracycline* | 20 | <95% | <95% | <95% |
|  | doxycycline** | 20 | <95% | <95% | <95% |
|  | oxytetracycline | 20 | <95% | <95% | <95% |
|  | chloretetracycline | 20 | <95% | <95% | <95% |
| Beta-lactams | amoxicillin** | 20 | <95% | <95% | <95% |
|  | ampicillin* | 20 | <95% | <95% | <95% |
| Fluoroquinolones | ciprofloxacin** | 20 | <95% | <95% | <95% |
|  | danofloxacin | 15 | <95% | <95% | <95% |
|  | enrofloxacin | 20 | <95% | <95% | <95% |
|  | norfloxacin | 20 | <95% | <95% | <95% |
| Sulfonamides | sulfisoxazole | 20 | <95% | <95% | <95% |
|  | sulfachloropyrazine | 15 | <95% | <95% | <95% |
|  | sulfachloropyridazine | 20 | <95% | <95% | <95% |
|  | sulfadiazine | 15 | <95% | <95% | <95% |
|  | sulfadimidine | 20 | <95% | <95% | <95% |
|  | sulfadoxine | 20 | <95% | <95% | <95% |
|  | sulfadimethoxine | 20 | <95% | <95% | <95% |
| Macrolides | Tylosin | 20 | <95% | <95% | <95% |
|  | Tilmicosin | 20 | 43.62 | 56.71 | 39.83 |
|  | Erythromycin* | 20 | <95% | <95% | <95% |
| Coccidiostates | Salinomycin | 20 | <95% | <95% | <95% |
|  | Narasin | 20 | 42.44 | 57.19 | 54.98 |
|  | Diclazuril | 20 | 56.13 | 82.70 | 46.97 |
|  | Clopidol | 20 | 32.69 | 42.88 | 36.45 |
|  | Niacrbazin | 20 | 56.63 | 51.52 | 79.06 |
|  | Maduramicin ammoniom | 20 | 82.01 | 82.42 | 77.41 |
|  | Monensin sodium | 20 | 92.42 | 95.82 | 73.56 |
|  | Robenidine hydrochloride | 20 | 48.35 | 34.30 | 44.03 |
|  | Decoquinate | 20 | 152.03 | 148.58 | 130.00 |

* Critical antimicrobials

** highly important antimicrobials
